# Influence of *Rapana venosa* Protein Hydrolysate on the Mechanical and Optical Performance of Polysaccharide-Based Composite Films

**DOI:** 10.3390/polym18070820

**Published:** 2026-03-27

**Authors:** Neslihan Akyurt, Koray Korkmaz

**Affiliations:** 1 Department of Food Processing, Espiye Vocational School, Giresun University, 28600 Giresun, Turkey; neslihanyolasgmazoglu@gmail.com; 2 Department of Fisheries Technologies, Fatsa Faculty of Marine Sciences, Ordu University, 52400 Ordu, Turkey

**Keywords:** biodegradable packaging films, *Rapana venosa* protein hydrolysate, optical and mechanical properties, SWARA, EDAS, sustainability

## Abstract

In this study, a multicomponent composite film system based on alginate, chitosan, κ-carrageenan, agar, and *Rapana venosa* protein hydrolysate (RVPH) was developed, and the effect of RVPH incorporation (0–1.5%) on molecular interactions, microstructure, and functional performance was evaluated using FTIR, SEM, mechanical testing, optical analysis, and water-related measurements. FTIR results indicated that RVPH interacted with the polysaccharide matrix mainly through hydrogen bonding and ionic interactions without causing chemical degradation. SEM analysis revealed concentration-dependent microstructural changes, with smoother morphologies at low RVPH levels and increased roughness and heterogeneity at higher concentrations. These structural differences were reflected in the functional properties. All films exhibited high swelling and water solubility. Optical properties were significantly affected by RVPH. Mechanical properties exhibited a non-linear response, with numerical variations observed but no statistically significant differences (*p* > 0.05). The EDAS and SWARA methods were employed to determine the optimal incorporation level of RVPH in the film formulations. Among the RVPH-containing films, the formulation incorporating 1% RVPH was identified as the most suitable alternative.

## 1. Introduction

The environmental impacts of conventional petroleum-based packaging materials, together with increasing global sustainability pressures, have intensified research efforts toward biodegradable and edible packaging systems [1]. In food lining applications, there is a growing demand for alternative materials that are not only environmentally benign but also capable of providing adequate barrier performance and functional stability under practical conditions. Within this context, polysaccharide-based films have attracted considerable attention due to their renewable origin, biocompatibility, and inherent film-forming ability [2]. However, single-component biopolymer films rarely meet the combined mechanical, optical, and water-related performance requirements demanded by food packaging applications.

Polysaccharides such as alginate, chitosan, κ-carrageenan, and agar are widely used in biodegradable film systems because of their distinct charge characteristics, gel-forming mechanisms, and ability to participate in diverse intermolecular interactions [3]. Despite these advantages, each polymer exhibits inherent limitations, including high water sensitivity, limited mechanical strength, or reduced structural stability when used individually [4]. Consequently, multicomponent polysaccharide film systems have been widely explored as a strategy to improve functional performance through polyelectrolyte complexation and synergistic network formation. Nevertheless, although combining multiple polysaccharides is a well-established concept, controlling the balance between matrix cohesion, flexibility, optical behavior, and water resistance in such complex systems remains a significant challenge [5].

Within this framework, protein hydrolysates have increasingly been incorporated into biopolymer films as functional modifiers rather than primary structural components. Due to their relatively low molecular weight and amphiphilic nature, protein hydrolysates can interact with polysaccharide matrices through various non-covalent mechanisms, thereby influencing network organization and macroscopic film properties [6]. Previous studies have shown that protein hydrolysates derived from different sources, including fish, gelatin, and plant proteins, can be integrated into polysaccharide-based matrices such as chitosan, alginate, or starch films, where they affect the mechanical, barrier, and structural properties of the resulting materials [7,8]. In such systems, protein hydrolysates may function as plasticizers or network modifiers by participating in hydrogen bonding, electrostatic interactions, and other non-covalent interactions with polysaccharide chains [9,10].

Despite the increasing use of protein hydrolysates in biopolymer films, their behavior within complex multicomponent polysaccharide matrices remains insufficiently explored. Although alginate–chitosan–κ-carrageenan–agar systems and marine protein hydrolysates have each been investigated individually, their combined behavior within a single multicomponent system has not been comprehensively clarified. Most previous studies have focused on relatively simple polymer matrices composed of one or two biopolymers, whereas the influence of protein hydrolysates on highly interactive multicomponent polysaccharide networks remains poorly understood [11,12,13]. In particular, limited attention has been given to how protein hydrolysate incorporation modifies an already established polyelectrolyte complex rather than forming an entirely new polymer matrix. This distinction is especially relevant for systems containing multiple charged polysaccharides, where subtle changes in interaction density may produce non-linear effects on mechanical, optical, and water-related properties. Moreover, the matrix investigated in this study is predominantly composed of marine-derived biopolymers—including alginate, κ-carrageenan, agar, chitosan, and *Rapana venosa* protein hydrolysate—forming a marine-based composite system whose interaction behavior may differ from that of commonly studied biopolymer films derived from terrestrial sources.

Accordingly, the objective of this study is to investigate the role of *Rapana venosa* protein hydrolysate (RVPH) as a functional network modifier within a multicomponent alginate–chitosan–κ-carrageenan–agar polysaccharide matrix. By combining FTIR spectroscopy, SEM analysis, mechanical testing, optical characterization, and water-related property evaluation, this work aims to clarify how RVPH influences molecular organization, microstructure, and structure–property relationships in a highly interactive polysaccharide network. In this way, the study contributes to a more comprehensive understanding of protein hydrolysate–polysaccharide interactions and supports the rational design of multicomponent biodegradable film systems for functional food lining applications.

## 2. Materials and Methods

### 2.1. Materials

Biopolymers based on chitosan, carrageenan, agar, alginate, and protein hydrolysate were selected for film production. The polysaccharide-based components forming the film matrix consisted of chitosan, carrageenan, agar, and alginate, while glycerol was used as a plasticizer in the film formulations. Deionized water was used in all film-forming solutions.

Low-molecular-weight chitosan (degree of deacetylation: 75–85%; molecular weight: 50,000–190,000 Da; viscosity: 20–300 cP for a 1 wt.% solution) was purchased from Sigma-Aldrich (St. Louis, MO, USA) and used without further purification. Low-molecular-weight chitosan was selected due to its improved solubility and higher chain mobility, which facilitates more homogeneous dispersion and intermolecular interaction within multicomponent polysaccharide film-forming matrices.

Carrageenan (E407) was obtained from Calmotex, South Korea; supplied by Benosen, Istanbul, Turkey. According to the supplier specifications, carrageenan exhibited a gel strength of 1800 g/cm^2^ in water (1.5% gel at 20 °C) and 780 g/cm^2^ in KCl solution (0.6% gel, 0.2% KCl at 20 °C). KCl was purchased from Kimyalab (Istanbul, Turkey). Agar was purchased from Dr. Gusto (Istanbul, Turkey). Sodium alginate (Chem Pure, viscosity grade: 1000) was supplied by Zag Kimya (Istanbul, Turkey). Glycerol (99.7% purity) supplied by Kimyacınız (Istanbul, Turkey) was used as a plasticizer in the film formulations.

The raw material used for protein hydrolysate production was obtained from *Rapana venosa* specimens sourced from a commercial supplier. The samples were transported to the laboratories of the Faculty of Marine Sciences, Ordu University, under cold-chain conditions and stored at −40 °C until further processing for protein hydrolysate production.

Protein hydrolysate was incorporated into the film formulations at concentrations ranging from 0 to 1.5%.

### 2.2. Preperation of Rapana venosa Protein Hydrolysate

Protein hydrolysate production was carried out based on the enzymatic hydrolysis method optimized by Korkmaz and Tokur [14]. Frozen *Rapana venosa* meat stored in 1 kg packages was thawed under controlled cold conditions (0–4 °C) and mechanically minced using a meat grinder (Empero, EMP.12.01.P, Konya, Turkey). The minced samples were heated in a water bath (Memmert WNB 22, Schwabach, Germany) (at 90 °C for 20 min to suppress endogenous enzyme activity. Following heat treatment, the samples were allowed to cool to room temperature.

After cooling, distilled water was added to the samples at a 1:1 (*w*/*w*) ratio, and the mixture was homogenized using an Ultra-Turrax (T25, IKA, Staufen, Germany) until a uniform suspension was obtained. Enzymatic hydrolysis was then carried out by adding commercially available Alcalase enzyme (Novozymes A/S, Bagsvaerd, Denmark) to the homogenized mixture. Hydrolysis was conducted at an enzyme concentration of 1% (*w*/*w*) at 50 °C for 1 h.

Upon completion of the hydrolysis process, the enzyme activity was terminated by heating the mixture at 85 °C for 10 min, followed by cooling for 15 min. To separate the resulting phases, the hydrolysate was centrifuged at 4000× *g* for 20 min (Sigma 3–30 KS, Darmstadt, Germany). The supernatant was subsequently freeze-dried, and the resulting protein hydrolysates were stored at 4 °C until further analyses.

### 2.3. Degreee of Hydrolysis (%HD)

The degree of hydrolysis (DH, %) was calculated according to the method proposed by Hoyle and Merritt [15]. Briefly, after completion of the hydrolysis process, the samples were mixed with 20% (*w*/*w*) trichloroacetic acid (TCA) solution at a 1:1 (*v*/*v*) ratio. The resulting mixture was centrifuged at 15,000× *g* for 20 min at 4 °C.

Following centrifugation, the amount of protein soluble in 10% TCA present in the clear supernatant was determined. All analyses were performed in triplicate. The degree of hydrolysis was calculated based on the ratio of TCA-soluble protein to the total protein content of the hydrolysate.

The degree of hydrolysis (DH, %) was expressed using the following Equation (1):

(1) $$\%DH=(N_{o}/Nt)\times 100$$

where N_o_ represents the amount of protein soluble in 10% TCA, and Nt denotes the total protein content of the hydrolysate.

### 2.4. Proxymate Composition

The proximate composition of the raw material and the resulting protein hydrolysates was determined. Moisture content was measured using a gravimetric method based on drying the samples in an oven at 105 °C until constant weight was achieved [16]. Protein content was calculated after determination of total nitrogen by the Kjeldahl method, using a nitrogen-to-protein conversion factor of 6.25 [17]. Lipid content was analyzed according to the Soxhlet extraction method [18]. Ash content was determined by a gravimetric method based on incineration of the samples in a furnace at temperatures ranging from 500 to 600 °C [19].

### 2.5. Preperation of Biodegradable Composite Films

The composition of the composite film formulations used in this study is summarized in Table 1. The film matrix consisted of chitosan, agar, κ-carrageenan, and sodium alginate, while glycerol was used as a plasticizer and *Rapana venosa* protein hydrolysate (RVPH) was incorporated as a functional additive. While the concentrations of the film-forming components are provided in Table 1, the preparation procedure of the film-forming solutions is described below. The polysaccharide matrix composition and processing conditions were kept constant in all formulations, while only the concentration of *Rapana venosa* protein hydrolysate (RVPH) was varied.

Biopolymer-based composite films were prepared using the casting technique by combining and modifying the methods previously reported by Sogut et al. [20], Zhao et al. [21], and Fıçıcılar and Korkmaz [22]. Film-forming solutions were prepared using a sequential mixing approach under magnetic stirring. First, First, a 0.5% (*w*/*v*) sodium alginate solution (30 mL) was prepared by dissolving alginate in distilled water under magnetic stirring until complete dissolution was achieved. Separately, a 2% (*w*/*v*) chitosan solution (100 mL) was prepared by dissolving chitosan in 1% (*v*/*v*) acetic acid solution under continuous stirring until a clear solution was obtained. For protein-containing formulations, RVPH was added to the chitosan solution at different concentrations (0, 0.5, 1.0, and 1.5%, *w*/*w*), and the mixtures were stirred until a homogeneous dispersion was obtained.

In another vessel, 0.30 g agar and 0.15 g κ-carrageenan were dissolved in 70 mL distilled water under controlled heating (approximately 90 °C) to ensure complete dissolution. This corresponded to an agar/κ-carrageenan ratio of approximately 2:1 in the base polysaccharide matrix. The solution was then allowed to cool to 45–50 °C, after which the previously prepared chitosan (or RVPH–chitosan) solution was added under continuous magnetic stirring.

Following the incorporation of chitosan, glycerol was added as a plasticizer at a concentration of 2.5% (*w*/*w*) based on the total polymer content. The mixture was then cooled further to approximately 20–30 °C, and the alginate solution was added as the final component of the film-forming system. All mixing steps were carried out under magnetic stirring to ensure complete homogeneity of the film-forming solutions.

**Figure 1:**
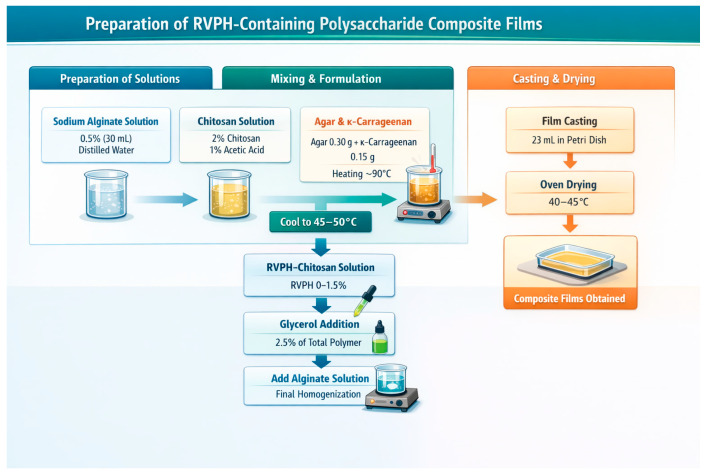
Schematic representation of the preparation procedure of polysaccharide-based composite films containing *Rapana venosa* protein hydrolysate (RVPH).

After complete homogenization, the solutions were allowed to stand at room temperature for approximately 5 min to promote initial gel formation and eliminate air bubbles. Subsequently, 23 mL of the film-forming solution was cast onto rectangular Petri dishes (127.8 mm × 85.5 mm) and dried in an oven at 40–45 °C until completely dried to obtain the composite films. The overall preparation procedure is illustrated schematically in Figure 1.

### 2.6. Film Characterization

#### 2.6.1. Moisture Content Determination

The moisture content of the films was determined using a gravimetric method. For this purpose, film samples were initially weighed and then dried in an oven at 105 °C until a constant weight was achieved. During the drying process, the samples were weighed at regular intervals, and the point at which the difference between successive measurements became negligible was considered as constant weight.

Moisture content was calculated based on the difference between the sample weights before and after drying and expressed as a percentage (%). All measurements were performed in at least three replicates.

#### 2.6.2. Film Thickness Measurement

The thickness of the films was measured using a digital micrometer. For each film sample, thickness measurements were taken at four different positions to ensure representative homogeneity, and the average of these values was reported as the film thickness. The digital micrometer used in this study had a measurement accuracy of 0.001 mm. All measurements were conducted at room temperature.

#### 2.6.3. Swelling Index

The swelling behavior of the films was evaluated according to the method reported in the literature [19]. Prior to analysis, film samples were dried to constant weight, and their initial dry weights were recorded using an analytical balance. The weighed film samples were immersed in beakers containing 50 mL of distilled water and placed in a shaker operating at 100 rpm and 25 °C for 30 min.

At the end of the predetermined period, the films were removed from the water, and excess surface water was gently blotted using filter paper. The swollen films were then reweighed. The swelling index was calculated based on the ratio of the amount of water absorbed to the total dry mass of the film and expressed as g water absorbed per g of dry film.

#### 2.6.4. Water Solubility (WS)

The water solubility of the films was determined using a gravimetric method. Film samples were cut into 2 cm × 2 cm pieces, and their initial dry weights were recorded using an analytical balance. To ensure complete drying, the samples were dried in an oven at 100 °C for 24 h, and their constant dry weights were determined.

After drying, the film samples were immersed in 15 mL of distilled water at room temperature and kept under continuous agitation for 24 h. At the end of the immersion period, the insoluble film residues were separated from the solution using Whatman filter paper. The remaining insoluble fraction was subsequently dried again, and the final dry weight was recorded.

The percentage water solubility of the films (*WS*%) was calculated based on the difference between the initial dry weight and the final dry weight after immersion, using Equation (2):

(2) $$WS\left(\%\right)=\frac{W_{i}-W_{f}}{W_{i}}\times 100$$

where *W*_*i*_ is the initial dry weight (mg) and *W*_*f*_ is the final dry weight (mg).

#### 2.6.5. Color Measurement

The color properties of the films were evaluated using a colorimeter (Konica Minolta CM-5, Osaka, Japan) based on the CIELab color system, recording L* (lightness), a* (red–green axis), and b* (yellow–blue axis) parameters. Film samples were placed over the measurement aperture of the instrument, and color measurements were taken at different positions on each sample to account for surface heterogeneity. The average L*, a*, and b* values were calculated and used to characterize the color properties of the films.

#### 2.6.6. Opacity

The opacity of the films was determined using a UV–Vis spectrophotometer. Measurements were performed at a wavelength of 600 nm using a UV–Vis spectrophotometer (UV-1280, Shimadzu, Kyoto, Japan). For each film sample, the absorbance value recorded at 600 nm was divided by the film thickness to calculate the opacity value.

#### 2.6.7. Mechanical Testing

The mechanical properties of the films were evaluated in terms of tensile strength (TS), elongation at break (EB), and toughness. Mechanical tests were performed using a texture analyzer (Stable Micro Systems, Surrey, UK) equipped with tensile grips (A/TG probe), and data acquisition and analysis were conducted using Exponent software (version 6.1.16.0). All measurements were carried out at room temperature (approximately 23 ± 2 °C) under ambient laboratory humidity conditions in accordance with the ASTM D882-18 [23] standard test method.

Rectangular film specimens (15 mm × 70 mm) were mounted between the tensile grips with an initial gauge length of 40 mm. During the tests, the maximum recommended load capacity of the instrument (50 kg) was applied. Tensile tests were conducted at a crosshead speed of 1.00 mm/s during the test phase, followed by a post-test crosshead speed of 10.00 mm/s.

#### 2.6.8. FTIR Analysis

The functional group structures of the film samples were analyzed using a Fourier Transform Infrared (FTIR) spectrometer equipped with an Attenuated Total Reflectance (ATR) accessory (PerkinElmer Spectrum Two, Waltham, MA, USA). FTIR spectra were recorded in the wavenumber range of 600–4000 cm^−1^ at a resolution of 4 cm^−1^. To improve spectral reliability, four scans were collected for each sample, and the resulting spectra were evaluated.

#### 2.6.9. SEM Analysis

The surface morphology of the films was examined using a scanning electron microscope (SEM). SEM imaging was performed using a HITACHI SU1510 instrument (Tokyo, Japan). To evaluate the morphological characteristics of the films, the upper surfaces were observed at magnifications of 100× and 1000×, while the lower surfaces were examined at a magnification of 1000×. The obtained SEM micrographs were used for qualitative assessment of surface structure, homogeneity, and morphological integrity of the films.

#### 2.6.10. Statistical Analysis

All experiments were conducted in triplicate. Differences among film samples were evaluated using one-way analysis of variance (ANOVA), and Tukey’s multiple comparison test was applied to identify statistically significant differences between groups. Statistical analyses were performed using SPSS software (version 18.0; SPSS Inc., Chicago, IL, USA), and the level of statistical significance was set at *p* < 0.05, corresponding to a 95% confidence interval.

#### 2.6.11. Application of MCDM Methods

As evidenced by the water-related, optical, mechanical, surface (SEM), and FTIR analyses of the polysaccharide-based composite films, the optimal formulation ratio varied according to the evaluated property. While a given formulation performed better in one set of measurements, another formulation exhibited more favorable responses in different analyses. Therefore, to identify the optimal incorporation level of *Rapana venosa* protein hydrolysate within the multi-polysaccharide matrix through a comprehensive evaluation of all relevant performance criteria, the SWARA and EDAS multi-criteria decision-making approaches were employed. These methods are frequently used, particularly in solving complex decision-making problems [24].

##### SWARA Method

The SWARA method is one of the MCDM methods used to address uncertainties in the process of evaluating criteria and alternatives through linguistic expressions [25]. The main advantage of the SWARA method in decision-making problems is that it enables the determination of criterion priorities based on business strategies, organizational plans, or national policies without requiring an additional evaluation to rank the criteria [26].

The steps followed in the SWARA method are as follows:

The following steps are followed to determine the weights of the criteria using the SWARA method [27].

Step 1. The criteria are ranked from highest to lowest based on expert opinions. If more than one expert participates in the evaluation, the criteria are first ranked individually by each expert. Then, the geometric mean of the individual rankings is calculated to obtain the final ranking of the criteria.

Step 2. The relative importance level of each criterion is determined by the relevant parties. To do this, criterion *j* is compared with the criterion that follows it (*j* + 1) to determine how much more important it is than the criterion being compared [28].

Step 3. The *kj* coefficient is determined as follows:

(3) $$kj=\left\{\begin{array}{ll} 1 & j=1\\ sj+1 & j>1 \end{array}\right.$$

Step 4. The *qj* variable is calculated as follows:

(4) $$qj=\left\{\begin{array}{ll} 1 & j=1\\ qj-1/kj & j>1 \end{array}\right.$$

Step 5. The relative weights of the evaluation criteria, *wj*, determine the relative weight of criterion *j*.

(5) $$wj=\frac{qj}{\sum_{k=1}^{n}qk}$$

When calculations are performed according to the SWARA method, the factors considered in solving the problem are ranked by experts from the most important to the least important. In cases where there is more than one decision-maker involved in solving the problem, each decision-maker first creates an individual ranking of the criteria. These rankings are then arranged from the least important to the most important, and their geometric means are calculated to obtain an overall ranking. After the ranking process, the relative importance weights of each factor are determined in comparison with the factor that follows it. The factors are ordered from the most important to the least important as: 1,2,3, …, *n* and the relative importance level of each criterion is determined [29]. In decision problems that include a large number of criteria, the SWARA method requires fewer pairwise comparisons compared to other methods. For a decision problem containing *n* criteria *n* − 1 comparisons are sufficient [27]. Based on these evaluations, the relative importance coefficient (*s_i_*) for each criterion is calculated. In the next step, the coefficient values (*k_i_*) are determined, and by using these coefficients, the recalculated weights of the criteria (*q_i_*) are obtained step by step. In the final stage, all criterion weights are normalized to determine the final weight values (*w_i_*).

##### EDAS Method

EDAS is one of the MCDM methods introduced to the literature by Ghorabaee and colleagues in 2015. The EDAS method bases its evaluations on the distances from the average solution to determine the most suitable alternative in the decision-making process [30].

The steps followed in the EDAS method are as follows [30].

Step 1. Creating the Decision Matrix (X): The decision matrix is shown in Equation (6) below. In the relevant matrix, X*ij* represents the performance value of alternative j according to criterion j.

(6) $$X=Xij=\left[\begin{array}{cccc} a11 & a12 & \ldots & a1n\\ a21 & a22 & \ldots & a2n\\ \vdots & \vdots & \ddots & \vdots \\ am1 & am2 & \ldots & amn \end{array}\right]$$

Step 2. In the second stage of the EDAS method, the average values matrix for the evaluation criteria is determined using Equation (5).

(7) $$\mathrm{AV}j=[\frac{\sum_{i=1}^{n}Xij}{n}]$$

Step 3. Creating the Positive and Negative Distance Matrices from the Average: In the EDAS method, a Positive Distance from Average (PDA) matrix and a Negative Distance from Average (NDA) matrix are constructed for each criterion. At this stage, the performance of each alternative with respect to the relevant criterion is compared with the corresponding average value.

(8) $$\mathrm{PDA}=\left[PDAij\right]nxm$$

(9) $$PDAij=[\frac{\max\left(0,\left(Xij-AVj\right)\right)}{AVj}],\;J\in \mathrm{Benefit}\;\mathrm{Value}$$

(10) $$NDAij=[\frac{\max\left(0,\left(AVj-Xij\right)\right)}{AVj}],\;J\in \mathrm{Benefit}\;\mathrm{Value}$$

(11) $$PDAij=[\frac{\max\left(0,\left(AVj-Xij\right)\right)}{AVj}],\;J\in \mathrm{Cost}\;\mathrm{Value}$$

(12) $$NDAij=[\frac{\max\left(0,\left(Xij-AVj\right)\right)}{AVj}],\;J\in \mathrm{Cost}\;\mathrm{Value}$$

Step 4. Calculation of Weighted Total Values: In the EDAS method, the Weighted Total Positive Distance (SP*i*) and Weighted Total Negative Distance (SN*i*) values for each alternative are calculated using Equations (12) and (13), respectively.

(13) $$SPi=[\sum_{j=1}^{m}wj\times PDAij]$$

(14) $$SNi=[\sum_{j=1}^{m}wj\times NDAij]$$

Step 5. In Step 5 of the EDAS method, the weighted and normalized values for all alternatives are calculated using Equations (14) and (15), respectively.

(15) $$NSPi=[\frac{SPi}{maxi\left(SPi\right)}]$$

(16) $$NSNi=[1-\frac{SNi}{maxi\left(SNi\right)}]$$

Step 6. Calculation of Success Scores for Each Alternative: In the final stage of the EDAS method, the success score to be used in the performance evaluation is obtained by taking the average of the values calculated for each alternative in the previous stage.

(17) $$Asi=[\frac{1}{2}\left(NSPi+NSNi\right)]$$

When applying the EDAS method, the decision-making process benefits from evaluations based on the distance from the average solution to determine the most optimal alternative [30]. According to this method, calculations begin with the construction of a decision matrix consisting of alternatives and criteria. In this matrix, each alternative is evaluated according to the relevant criteria. Then, the average values for each criterion are calculated to determine the average solution. In the next step, for each alternative, the Positive Distance from Average (PDA) and Negative Distance from Average (NDA) values are calculated for each criterion. These values indicate how much better or worse each alternative performs relative to the average solution. The calculated PDA and NDA values are then weighted by multiplying them with the corresponding criterion weights. Subsequently, the weighted PDA and NDA values are normalized. In the final stage, the normalized positive and negative distance values are combined to obtain the evaluation scores of the alternatives. According to the EDAS method, the alternative with the highest evaluation score is identified as the best alternative [31].

The advantages of the methods selected within the scope of the study over single parameter optimization approaches are as follows:

Since the SWARA method focuses on determining the relative importance of criteria, it provides a more systematic and expert opinion-based weighting even when there are many criteria in decision problems. While traditional single-parameter methods typically focus on a single objective, SWARA evaluates the importance of all criteria step by step and transparently in multi-criteria decision problems. Furthermore, since only *n* − 1 pairwise comparisons are required for n criteria, the computational load is lower compared to other multi-criteria methods [32].

According to the EDAS method, the performance of alternatives is evaluated based on their distance from the average solution. Unlike traditional single parameter optimization, this approach considers all criteria of the decision problem to reveal the relative superiority of alternatives. Calculating and weighting the positive and negative distances of alternatives and then normalizing them makes multi criteria performance evaluation more objective and comparable. Thus, the alternative with the highest score is determined as the optimal choice from a multi criteria perspective [33]. The criterion weights were calculated using the SWARA method. In the subsequent stage, the EDAS method was applied to determine the optimal protein ratio based on the calculated weights. For this purpose, opinions were gathered through a survey from a group of five (5) experts directly related to the subject. The identified factors and evaluated alternatives are presented in detail in Table 2.

## 3. Results and Discussion

### 3.1. Characterization of Rapana venosa Protein Hydrolysate

**Figure 2:**
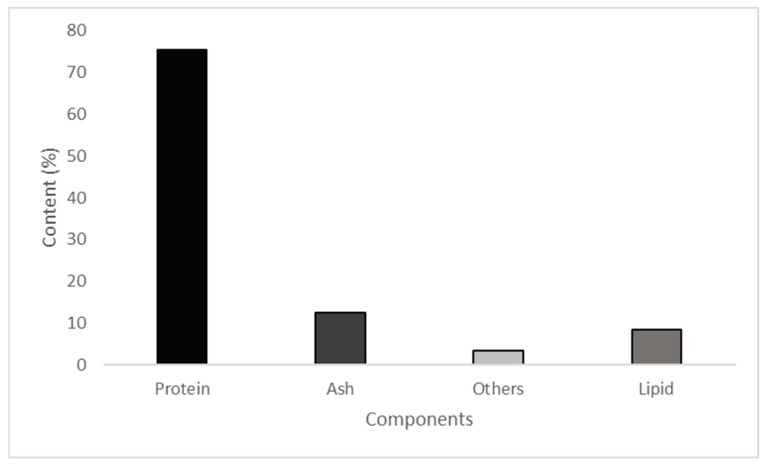
Proxymate composition of *Rapana venosa* protein hydrolysate.

The proximate composition of *Rapana venosa* protein hydrolysate (RVPH) is illustrated in Figure 2. The hydrolysate exhibited a high protein content (75.5%) together with a low lipid level (3.4%), indicating that the applied enzymatic hydrolysis and subsequent separation steps were effective in producing a protein-enriched fraction with limited fat content. The ash content of RVPH was determined to be 12.6%, which can be attributed to the naturally high mineral composition of marine-derived raw materials. The remaining fraction, consisting of moisture and residual carbohydrates, accounted for only a minor proportion of the hydrolysate. Overall, the compositional distribution presented in Figure 1 highlights the protein-rich nature of RVPH and supports its suitability as a functional additive for composite film formulations.

In addition to compositional characteristics, the degree of hydrolysis (DH) is an important indicator of peptide bond cleavage and plays a critical role in determining the interaction potential of protein hydrolysates with polysaccharide matrices. In the present study, the DH of RVPH was determined to be 55.5%, indicating extensive enzymatic degradation of muscle proteins into shorter peptide chains.

### 3.2. Film Thickness

The mean film thickness (mm) values of the RVPH-incorporated formulations are presented in Table 3.

The thickness of the composite films prepared in this study ranged between 0.054 and 0.178 mm. The incorporation of *Rapana venosa* protein hydrolysate (RVPH) resulted in statistically significant differences in film thickness among the formulations (*p* < 0.05). In contrast, Fıçıcılar and Korkmaz [22] and Oh et al. [34] reported that the inclusion of proteinaceous additives in biopolymer matrices did not cause significant changes in film thickness, particularly when applied at comparable concentration levels. Overall, the significant variations observed in the present study may be attributed to differences in matrix complexity, formulation strategy, or hydrolysate–polysaccharide interactions.

### 3.3. Moisture Content

**Figure 3:**
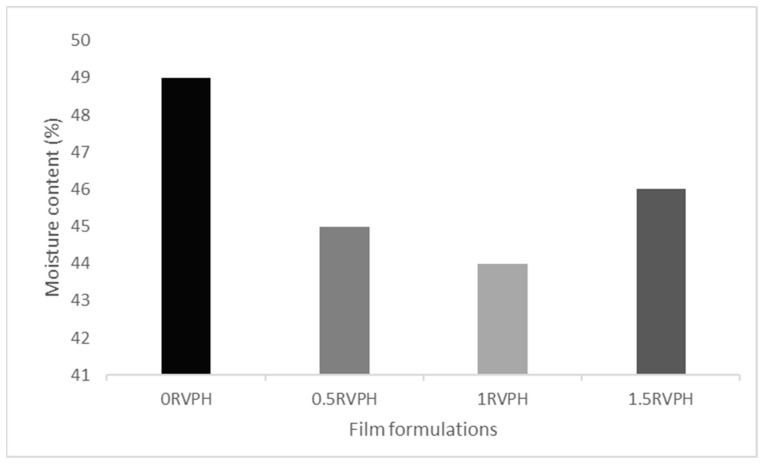
Moisture content (%) of composite films containing different concentrations of RVPH.

The moisture content of the prepared composite films ranged between 45% and 49%, and the corresponding results are presented in Figure 3.

When film formulations containing different RVPH concentrations were compared, similar moisture content values were obtained, and no statistically significant differences were observed among the groups (*p* > 0.05). Accordingly, within the investigated concentration range, RVPH incorporation did not lead to statistically distinguishable changes in the moisture content of the films. It is well documented that the moisture content of biopolymer-based films is largely governed by the hydrophilic nature of the constituent polymers, the abundance of water-interacting functional groups, and the overall water-holding capacity of the polymer network. Agar, in particular, is a polysaccharide with a high water-binding ability, and its water retention capacity has been reported to reach approximately 18 g/g [35]. Consistent with this behavior, Şahiner et al. [36] reported moisture contents ranging from 76% to 82% in agar–chitosan-based hydrogel films, attributing this to the physical entrapment of water within the three-dimensional agar gel network.

Similarly, high moisture contents have been reported for carrageenan-based film systems. Larotonda et al. [37] observed moisture contents between 55.6% and 83.3% in carrageenan films and attributed these values to the strong water affinity of the sulfate groups present in the carrageenan structure. Moreover, carrageenan has been shown to form physically crosslinked three-dimensional networks in the presence of suitable interactions, enabling the retention of substantial amounts of water while maintaining structural integrity [38].

Chitosan and glycerol, used as a plasticizer in the present study, are also recognized as important contributors to film moisture content. Bonilla et al. [39] reported that positively charged chitosan chains form extensive hydration layers, resulting in films with high water retention capacity. Similarly, Fundo et al. [40] demonstrated that increasing chitosan and glycerol concentrations led to higher moisture contents in biopolymer film matrices. Razavi et al. [41] reported moisture contents ranging from 27% to 49% in glycerol-plasticized films, depending on formulation parameters.

In this context, the moisture content values obtained for the alginate–chitosan–κ-carrageenan–agar–protein hydrolysate composite films fall within the range reported for comparable hydrophilic biopolymer systems. The absence of statistically significant variation with increasing RVPH concentration suggests that, under the conditions studied, the protein hydrolysate did not measurably alter the overall water-holding behavior of the composite matrix.

### 3.4. Swelling Index and Water Solubility

The influence of RVPH concentration on swelling ratio and water solubility is summarized in Table 4.

The control film (0 RVPH) exhibited the highest swelling ratio, reaching 7 g/g. This finding suggests that, in the protein-free matrix, interactions between polymer chains were relatively weaker and the network structure displayed a looser morphology. Such a structure may have facilitated the diffusion of water molecules into the matrix and their interaction with hydrophilic functional groups. In contrast, a significant decrease in swelling ratio was observed following the incorporation of RVPH. Statistical analysis indicated that the swelling ratio of the control film (0 RVPH) differed significantly from those of all RVPH-containing formulations (*p* < 0.05), whereas no significant differences were detected among the 0.5, 1, and 1.5 RVPH groups (*p* > 0.05). In particular, the swelling ratio decreased to 0.25 g/g in the film containing 0.5 RVPH, while comparable values of 0.375 and 0.5 g/g were observed at 1 and 1.5 RVPH concentrations, respectively.

This reduction may be attributed to the formation of additional hydrogen bonds and electrostatic interactions between polymer chains in the presence of the protein hydrolysate, resulting in a more compact network structure with lower free volume. The increased density of molecular interactions may have restricted chain mobility and thereby limited water penetration into the matrix. In addition, the limited water–glycerol exchange kinetics of glycerol entrapped within the film matrix may also have contributed to the suppression of swelling behavior [42]. Nevertheless, slight numerical variations in swelling ratio were observed when the RVPH concentration exceeded 0.5; however, these differences were not statistically significant (*p* > 0.05). This behavior may be associated with the increased number of free hydrophilic amino and carboxyl groups in the hydrolysate at higher concentrations, which could enhance its interaction capacity with water. However, the overall trend indicates that all RVPH-containing formulations exhibited lower swelling behavior compared with the control film.

The solubility values obtained in this study were higher than those reported for threadfin bream (*Nemipterus hexodon*)-based films, which contained soluble protein levels ranging from 55.61% to 79.22% [43]. Razavi et al. [41] reported that glycerol-plasticized films exhibit high moisture content (27–49%), high swelling capacity (110–140%), and approximately 80% water solubility. Orliac et al. [42] stated that cross-linked proteins in edible films do not dissolve and that increased cross-linking density may influence glycerol retention within the film network, thereby affecting solubility behavior. In this context, the swelling values of the films reflect not only the water absorption capacity of the protein network but also the amount of glycerol transferred from the network to the immersion medium via osmosis. However, since the glycerol content was kept constant in all formulations in this study, the observed differences are considered to primarily result from the modifying effect of RVPH on the network structure.

When the water solubility values were examined, a concentration-dependent bidirectional effect was observed. The solubility of the control film was determined to be 87.5%, whereas the incorporation of 0.5 RVPH reduced the solubility to 73.5%. This result suggests that low-level hydrolysate incorporation increased the interaction density between polymer chains, strengthened network integrity, and thereby limited solubility. However, as the RVPH concentration increased, solubility rose again, reaching 90% and 92.3% in the 1 and 1.5 RVPH formulations, respectively. Water solubility values differed significantly among the formulations (*p* < 0.05). At higher concentrations, the presence of more short-chain peptides and free hydrophilic groups in the system may have enhanced interactions with water and increased the dispersion tendency of the matrix in aqueous environments [22].

Overall, RVPH incorporation reduced swelling ratio and water solubility at low concentrations by increasing network density; however, at higher concentrations, the hydrophilic character of the hydrolysate became dominant, leading to an increasing trend in both swelling and solubility values. In general, films with a high solubility percentage are used for preparing food coatings and linings, whereas those with lower solubility are preferred for food packaging applications [44]. Films with higher water solubility may provide advantages in applications where dissolution during consumption or use as biodegradable packaging material is desired [45]. These results indicate that RVPH may function as a network-modifying component at the molecular level within the film matrix and that determining the optimal concentration according to the intended application is critical.

### 3.5. Color Properties

The obtained color parameters are given in Table 5.

The color characteristics of the films were evaluated using the CIELab color system, where the L* parameter represents lightness ranging from 0 (black) to 100 (white), a* indicates the color position between green (negative values) and red (positive values), and b* describes the color axis from blue (negative values) to yellow (positive values).

The L* values of the prepared films ranged from 35.42 to 36.20. Although slight numerical differences in lightness were observed, no statistically significant differences were detected among the film formulations (*p* > 0.05). This indicates that, within the investigated concentration range, RVPH incorporation did not produce a statistically distinguishable effect on film brightness, while maintaining consistent and homogeneous lightness across all formulations. Fıçıcılar and Korkmaz [22] reported L* values ranging from 29.38 to 33.50 for composite films containing anchovy protein hydrolysate (0–3%), with the highest lightness observed in the control films. The different behavior observed in the present study may be attributed to variations in the protein hydrolysate source, concentration range, and intrinsic pigment composition, as well as to the more complex polysaccharide-based matrix employed, which may buffer color changes associated with protein incorporation.

The a* values of the films ranged between −0.13 and 0.01 and remained close to zero for all formulations, indicating that the films largely preserved color neutrality along the red–green axis. Although statistically significant differences were observed with increasing RVPH content (*p* < 0.05), the a* values remained within a narrow range around zero, suggesting only limited variation without a pronounced shift toward either red or green tones. In contrast, Fıçıcılar and Korkmaz [22] reported a progressive shift toward more negative a* values with increasing hydrolysate concentration, while de Morais Lima et al. [46] reported a* values between −2.02 and −1.87 for chitosan–xanthan gum films containing fish protein hydrolysate. These differences further highlight the role of matrix composition in modulating the color response to protein hydrolysate incorporation.

The b* values of the films varied between −0.40 and 0.90, indicating a subtle yet directional shift from bluish to yellowish tones with increasing RVPH concentration. Films without RVPH and those containing 0.5% hydrolysate exhibited negative b* values, whereas films containing 1% and 1.5% hydrolysate showed positive b* values, with statistically significant differences among formulations (*p* < 0.05). This consistent transition suggests a concentration-dependent contribution of RVPH to the chromatic balance of the films, likely associated with the intrinsic color characteristics of the protein hydrolysate.

Overall, the color analysis demonstrates that RVPH incorporation influences the chromatic attributes of the films in a controlled and gradual manner without causing abrupt or visually disruptive color changes. This behavior indicates that RVPH can be incorporated into multicomponent polysaccharide-based films while preserving optical uniformity, an important consideration for food lining applications where appearance consistency is required.

### 3.6. Opacity and Light Transmittance

The optical properties of the films are presented in Table 6.

The incorporation of RVPH had a pronounced and statistically significant effect on the optical behavior of the composite films (*p* < 0.05). The opacity value of the control film (0% RVPH) was calculated as 2.44 mm^−1^. Upon the addition of 0.5% RVPH, opacity increased to 3.63 mm^−1^, indicating a substantial reduction in light penetration through the film matrix. The highest opacity value was observed in films containing 1% RVPH (5.35 mm^−1^), which simultaneously exhibited the lowest light transmittance (40.01%). Consistent with the findings reported by Harper et al. [47], these results demonstrate that RVPH incorporation up to an intermediate concentration enhances light-blocking capacity by increasing optical density within the composite structure.

When the RVPH content was further increased to 1.5%, opacity decreased to 3.00 mm^−1^, accompanied by a corresponding increase in light transmittance to 47.2%. This non-linear response suggests that, beyond a certain concentration, RVPH alters the internal organization of the film matrix, likely modifying light-scattering pathways rather than continuously increasing optical density. Similar concentration-dependent and non-monotonic trends have been reported in biopolymer films containing protein hydrolysates or multifunctional additives, where excessive additive loading can lead to heterogeneous microstructures and irregular optical responses [48,49].

Regarding light transmittance, the control films exhibited the highest value (51.6%), whereas RVPH incorporation progressively reduced light transmission, reaching a minimum at 1% RVPH. This consistent reduction indicates that RVPH contributes to the formation of light-scattering and/or light-absorbing domains within the polysaccharide-based matrix. At the microstructural level, the presence of dispersed peptide-rich domains may increase refractive index heterogeneity within the film, thereby enhancing light scattering and reducing transparency. Such behavior has been widely reported in protein- or peptide-enriched biopolymer films, where increased absorbance and reduced transparency are attributed to enhanced matrix heterogeneity and refractive index contrast [22].

Overall, the optical results indicate that RVPH incorporation modulates film opacity and light transmittance in a concentration-dependent manner. Statistical analysis confirmed that the observed differences among the formulations were significant (*p* < 0.05), demonstrating that RVPH significantly influences light–matter interactions within the composite films. Consequently, the formulation containing 1% RVPH exhibited the most effective light-shielding behavior, which is advantageous for packaging applications involving light-sensitive food products, where controlled opacity rather than maximum transparency is often desirable.

### 3.7. Mechanical Properties

The mechanical properties of the composite films were evaluated by uniaxial tensile testing, and the tensile strength (TS), elongation at break (EB), and toughness values are summarized in Table 7. These parameters represent key indicators of a film’s resistance to mechanical stress, deformation capacity, and energy absorption behavior, all of which directly influence its suitability for packaging applications [50]. Protein- and/or polysaccharide-based hydrocolloid films are widely known to exhibit lower mechanical strength than conventional synthetic polymer films, primarily due to their hydrophilic nature and flexible polymeric networks [51,52]. Accordingly, biodegradable hydrocolloid films are not intended to replace petrochemical polymer-based packaging materials. Instead, they are typically used as internal layers or functional coatings designed to control moisture, aroma, and lipid migration within composite food systems or at the food–headspace interface [53]. Within this framework, the mechanical behavior of such films should be interpreted in terms of formulation-dependent changes rather than absolute strength values.

As shown in Table 7, RVPH incorporation influenced the tensile behavior of the films. The tensile strength of the control film (0% RVPH) was measured as 0.626 ± 0.135 N/mm^2^. The tensile strength of the film containing 0.5% RVPH was measured as 1.035 ± 0.440 N/mm^2^. However, further increases in RVPH concentration resulted in lower tensile strength values, with 0.601 ± 0.206 N/mm^2^ and 0.766 ± 0.342 N/mm^2^ recorded for films containing 1.0% and 1.5% RVPH, respectively. Although numerical differences were observed among the formulations, these variations were not statistically significant (*p* > 0.05), indicating that RVPH incorporation did not produce statistically detectable changes in tensile strength within the investigated concentration range. Therefore, these variations should be interpreted as formulation-related tendencies rather than statistically confirmed differences in mechanical performance.

Nevertheless, some formulation-dependent tendencies can be considered. At lower RVPH concentrations, the numerical increase in tensile strength may be associated with enhanced intermolecular interactions within the polymer network. In contrast, at higher concentrations, the observed variations may be related to increased peptide–peptide interactions and potential microphase separation, which could affect the continuity of the polymer network. This interpretation is supported by SEM observations, where increased surface heterogeneity and aggregated domains were detected at higher RVPH concentrations. Similar concentration-dependent tendencies have been reported in protein hydrolysate-incorporated chitosan films [54].

Elongation at break values also varied depending on RVPH concentration. The control film exhibited an elongation at break of 71.74 ± 5.98%, which decreased to 60.50 ± 2.00% after the addition of 0.5% RVPH. With increasing RVPH content, EB values of 67.01 ± 6.38% and 71.00 ± 5.56% were recorded for films containing 1.0% and 1.5% RVPH, respectively. However, these differences were not statistically significant (*p* > 0.05), indicating that RVPH concentration did not produce statistically detectable changes in elongation behavior. This trend suggests that RVPH may influence the flexibility of the polymer network by modifying intermolecular interactions and deformation behavior depending on concentration. These observations may be associated with formulation-dependent variations in intermolecular interactions and deformation behavior within the polymer network. Similar tendencies have been reported in previous studies describing the plasticizing and network-modifying effects of protein hydrolysates in biopolymer-based films [49].

Toughness values ranged from 0.056 to 0.089 MJ/m^3^, and no statistically significant differences were observed among the film formulations (*p* > 0.05), indicating that RVPH incorporation did not significantly alter the overall energy absorption capacity of the films. Nevertheless, films containing 0.5% and 1.5% RVPH exhibited slight numerical differences in toughness values compared with the control film. However, these differences were not statistically significant (*p* > 0.05). Ferreira et al. [55] emphasized that the mechanical properties of chitosan-based films can vary widely depending on several factors, including polymer composition, molecular weight, film preparation method, and conditioning conditions, which may explain the variability observed in toughness values.

### 3.8. FTIR

**Figure 4:**
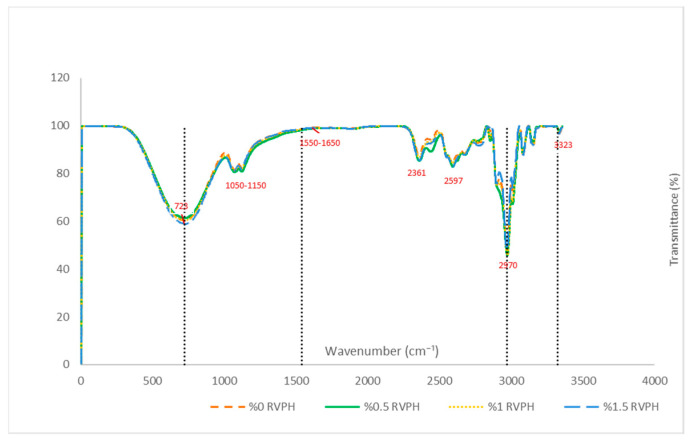
FTIR spectra of composite films containing different concentrations of *Rapana venosa* protein hydrolysate (RVPH). Vertical dashed lines indicate characteristic absorption regions corresponding to major functional groups discussed in the text.

The FTIR spectra of composite films containing different concentrations of *Rapana venosa* protein hydrolysate (RVPH) are presented in Figure 4.

The broad band observed in the 3200–3400 cm^−1^ region is attributed to overlapping O–H stretching vibrations of polysaccharides and glycerol, together with N–H stretching contributions originating from protein-derived amide groups. In multicomponent polysaccharide–protein film systems, extensive overlap of FTIR absorption bands is commonly observed because the constituent biopolymers contain similar functional groups, such as hydroxyl, amide, and ether groups. Therefore, the spectra are typically interpreted based on band intensity variations and slight shifts in characteristic regions rather than on the appearance of new peaks. Due to the multicomponent nature of the film matrix and the abundance of hydroxyl-rich polymers, this band primarily reflects collective hydrogen-bonding interactions within the system. With increasing RVPH content, slight variations in band intensity and width were observed, suggesting modifications in the hydrogen-bonding environment within the composite matrix. However, owing to the presence of multiple hydroxyl-rich components, these changes are interpreted as reflecting combined matrix interactions rather than RVPH-specific bonding, consistent with previously reported polysaccharide–peptide systems [49].

Bands detected in the 2800–3000 cm^−1^ region correspond to C–H asymmetric and symmetric stretching vibrations of aliphatic groups, primarily originating from polysaccharide backbones and glycerol. Comparable spectral profiles across all formulations indicate that RVPH incorporation did not induce pronounced alterations in aliphatic chain vibrations. This finding supports the assumption that the primary matrix framework remained dominated by polysaccharide components, while RVPH acted as a secondary contributor without disrupting the backbone structure.

In the amide I region (1700–1600 cm^−1^), associated mainly with C=O stretching vibrations of peptide bonds, gradual intensity changes were observed with increasing RVPH concentration. Although overlapping contributions from polysaccharide carbonyl groups limit definitive peak separation, the concentration-dependent variations are consistent with the incorporation of protein-derived structures into the composite films. These subtle intensity differences in the amide region further indicate the presence of protein-derived functional groups within the polysaccharide-dominated matrix. These observations indicate the presence of RVPH within the matrix rather than the formation of distinct protein domains.

Minor variations detected in the amide II region (approximately 1580–1500 cm^−1^), related to N–H bending and C–N stretching vibrations, further support the contribution of proteinaceous components to the overall spectral profile. The relatively low intensity and subtle nature of these changes suggest that RVPH was dispersed within the polysaccharide-dominated matrix rather than acting as a primary crosslinking agent, in agreement with previous observations in similar composite systems [56,57].

The bands observed at 1600–1625 cm^−1^ and 1410–1425 cm^−1^ correspond to the asymmetric and symmetric stretching vibrations of carboxylate (COO^−^) groups, characteristic of alginate and κ-carrageenan components. Changes in relative band intensities with increasing RVPH content suggest competitive ionic interactions within the polyelectrolyte system, particularly between carboxylate groups and protonatable amino groups present in chitosan and RVPH. Such concentration-dependent spectral variations reflect molecular rearrangements within the matrix rather than nonspecific spectral fluctuations and are consistent with previously reported multicomponent biopolymer systems [58,59,60].

The region between 1200–1000 cm^−1^, characteristic of C–O–C and C–O stretching vibrations in polysaccharides, displayed similar spectral patterns across all formulations. This observation confirms that the fundamental polysaccharide network structure was preserved upon RVPH incorporation and that the protein hydrolysate primarily influenced the matrix through secondary interactions rather than disrupting the polysaccharide backbone.

Additionally, the characteristic band observed in the 720–735 cm^−1^ region, attributed to the 3,6-anhydro-D-galactose ring vibrations of κ-carrageenan, was preserved in all formulations. The persistence of this band confirms that the structural integrity of the polysaccharide components remained intact following RVPH addition [61].

Overall, the FTIR results demonstrate that RVPH interacts with the alginate–chitosan–κ-carrageenan–agar matrix primarily through non-covalent interactions, including hydrogen bonding and electrostatic associations. Although the complexity of the multicomponent system precludes the isolation of RVPH-specific bonds, the systematic concentration-dependent spectral variations observed across increasing RVPH levels indicate that the protein hydrolysate contributes measurably to molecular organization within the composite films rather than acting as an inert filler. This interpretation provides a qualitative but internally consistent explanation for the role of RVPH within the composite matrix, in line with the accompanying mechanical and morphological observations.

### 3.9. Surface Morphology of Films

**Figure 5:**
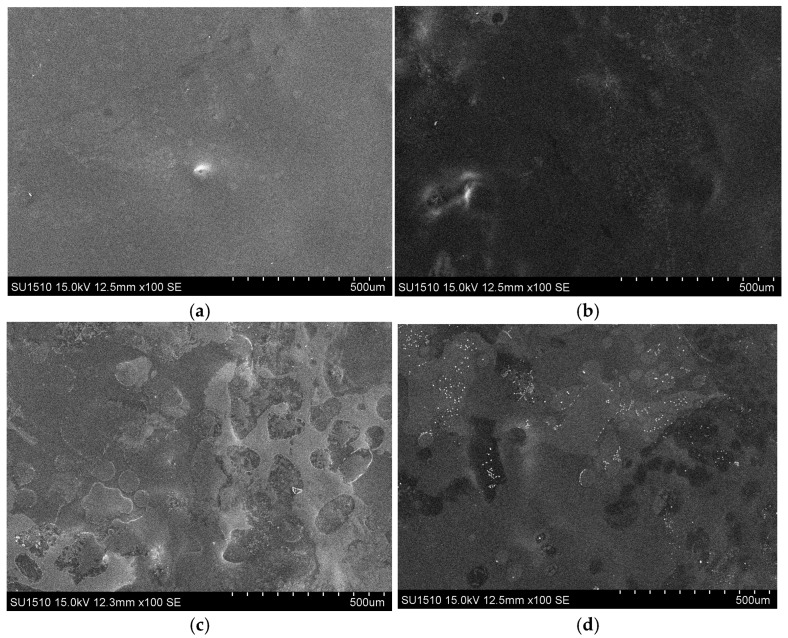
SEM micrographs of composite films containing different RVPH concentrations: (**a**) 0% RVPH, (**b**) 0.5% RVPH, (**c**) 1% RVPH, and (**d**) 1.5% RVPH, illustrating the effect of RVPH incorporation on surface morphology (magnification: ×100).

Figure 5 presents SEM micrographs of composite films containing different concentrations of *Rapana venosa* protein hydrolysate (RVPH). The surface morphology of the films was strongly dependent on RVPH content, exhibiting a clear transition from smooth and compact structures at low RVPH levels to more heterogeneous and irregular morphologies at higher concentrations.

As shown in Figure 5a, the control film (0% RVPH) displayed a smooth, continuous, and relatively compact surface, indicating the formation of a well-organized polymeric network dominated by the interactions among alginate, chitosan, κ-carrageenan, and agar. A similar morphological profile was observed for the film containing 0.5% RVPH (Figure 5b), which maintained surface homogeneity and structural continuity with only minor surface irregularities. These observations suggest that low RVPH incorporation does not significantly disturb the integrity of the polysaccharide-based matrix.

In contrast, films containing higher RVPH levels (1% and 1.5%) exhibited pronounced changes in surface topography. As shown in Figure 5c,d, these films displayed increased surface roughness, heterogeneous domains, microvoids, and irregular aggregated regions. Such morphological features indicate partial disruption of matrix uniformity, likely arising from intensified polymer–protein interactions and the limited compatibility of low-molecular-weight peptide fractions within the densely crosslinked polysaccharide network.

Similar morphological trends have been reported in alginate- and polysaccharide-based composite films incorporating functional additives. Torol and Ocak [62] observed increased surface roughness and phase heterogeneity in alginate films containing essential oils, which were attributed to weakened intermolecular cohesion and localized phase separation. Likewise, de Oliveira Filho et al. [63] reported that protein hydrolysate incorporation transformed alginate films from smooth and homogeneous structures into more heterogeneous morphologies due to protein aggregation and interference with polymer crosslinking regions.

In the present multicomponent system, the increased surface roughness observed at higher RVPH concentrations may be associated with disturbance of the polyelectrolyte complex equilibrium formed among chitosan, κ-carrageenan, and alginate. These polysaccharides are known to establish compact and cohesive networks through electrostatic interactions [64]. The introduction of RVPH likely alters local charge distribution and interaction density within the matrix, as low-molecular-weight peptides may compete with polysaccharide chains for ionic binding sites, resulting in localized aggregation and microphase separation.

The formation and stability of polyelectrolyte complexes in chitosan–κ-carrageenan–alginate systems are primarily governed by ionic interactions that promote dense and uniform network structures [65]. The incorporation of RVPH appears to partially perturb this balance, leading to less uniform surface features and the emergence of aggregated domains, particularly at higher concentrations. Comparable observations were reported by Khotimah et al. [66], who noted increased aggregation and surface irregularity with rising polysaccharide or additive content in composite film systems.

The surface properties of edible films, particularly smoothness and morphological homogeneity, play an important role in determining their functional performance in food coating and packaging applications. Porosity and microstructural irregularities formed on the film surface may influence gas diffusion pathways, potentially altering the permeability of oxygen and carbon dioxide [67]. Therefore, surface morphology is considered an important structural parameter that can affect the overall functional behavior of coating systems. Surface roughness and microporous structures observed in polysaccharide-based film systems containing chitosan are associated with the intrinsic morphological characteristics of this polymer. Such microporous structures may influence gas transfer behavior while also providing a suitable matrix for the transport and controlled release of active compounds [68]. The polymer concentration used in coating films is also regarded as an important parameter influencing surface morphology and, consequently, the functional behavior of the system. Coating systems with higher polymer content tend to exhibit a more compact and less porous microstructure, which may restrict gas diffusion and potentially trigger anaerobic respiration [69]. In contrast, coating films containing an appropriate number and size of micropores may regulate gas exchange to a certain extent, thereby forming a protective barrier capable of reducing decay, water loss, and respiration rate [68]. Therefore, controlling surface morphology is considered an important factor in regulating the microenvironment created by lining films at the food–packaging interface.

Overall, the SEM results clearly demonstrate that RVPH concentration plays a critical role in determining the microstructural organization of the composite films. Low RVPH levels preserve surface homogeneity and compactness, whereas higher concentrations induce structural heterogeneity and increased surface roughness. These morphological changes are consistent with FTIR findings indicating concentration-dependent intermolecular interactions, as well as with the non-linear mechanical behavior observed in the tensile properties. The structural heterogeneity and microvoid formation observed at higher RVPH concentrations may also explain the reduction in tensile strength observed in the mechanical tests. Disruption of the compact polysaccharide network and the formation of localized aggregated domains can reduce effective stress transfer within the film matrix, leading to decreased mechanical integrity at elevated RVPH levels. Collectively, these results confirm that RVPH incorporation governs the microstructural architecture of the multicomponent polysaccharide film system.

### 3.10. Determination of Optimal Protein Incorporation Level

Based on the SWARA analysis, the relative importance of the evaluation criteria was determined according to expert opinions. The calculated weights and overall ranking of the criteria are presented in Table 8 and obtained the priority vectors for the parameters in accordance with the SWARA method. As an example, the importance ranking derived from the comparisons made by the first decision maker (DM1) is presented in Table 9.

A detailed examination of the results indicates that the formulations exhibit parameter-dependent and, at times, contradictory performance profiles. The film containing 0.5% RVPH, for instance, displays comparatively higher tensile strength and toughness; however, it simultaneously exhibits the lowest swelling capacity, suggesting a trade-off between mechanical integrity and water-related behavior. Likewise, although the 1.5% RVPH formulation demonstrates relatively elevated toughness values, the pronounced phase separation observed in SEM analyses clearly points to structural instability, which compromises its overall functional performance.

In contrast, the remaining formulations do not consistently dominate across the evaluated properties, nor do they present uniformly poor performance. Rather, each formulation performs favorably in certain aspects while remaining limited in others, preventing a straightforward identification of an optimal system based on any single parameter.

Under these conditions, reliance on individual mechanical indicators alone would be insufficient and potentially misleading, as apparent improvements in one property may coincide with deterioration in others. Therefore, the SWARA/EDAS-based MCDM framework was employed to systematically integrate all relevant performance criteria into a unified decision structure, enabling a more robust and balanced assessment of the formulations.

According to Table 10, “Mechanical performance” was identified as the most important criterion within the framework established by the experts. The overall ranking of the criteria was determined as C1 > C3 > C2 > C4.

The decision matrix for the EDAS Method is provided in Table 11 below.

Table 12 below represents the Average Positive Distance Matrix.

Table 13 represents the Average Negative Distance Matrix.

Table 14 below shows the Evaluation Scores and ASi.

According to Table 14, A1 (0 RVPH) was identified as the most preferable alternative based on the EDAS method incorporating the criterion weights derived from the SWARA approach. The overall ranking of the alternatives was established as A1 > A3 > A2 > A4. The fact that the control formulation (0 RVPH) ranked first indicates that, when all evaluated criteria were considered collectively, the incorporation of *Rapana venosa* protein hydrolysate (RVPH) did not improve the overall performance; rather, it tended to adversely affect the mechanical structure of the polysaccharide films.

It has been reported that protein–polysaccharide complexes may exhibit enhanced functional properties compared with protein- or polysaccharide-based systems alone; however, these properties are highly dependent on physicochemical parameters such as pH, ionic strength, protein–polysaccharide ratio, charge distribution of the components, and molecular weight [70]. The structural behavior of protein and polysaccharide polymers may manifest either as compatible interactions or as phase separation, depending on molecular-level organization. In such polymer systems, the delicate balance between attractive and repulsive forces may promote the formation of a controlled network structure, whereas weak interactions, excessive intermolecular forces, or restricted molecular mobility may lead to phase separation and structural instability [9].

Accordingly, the lowest performance observed for the formulation containing 1.5% RVPH may be attributed to the pronounced phase separation detected in the SEM analyses, which likely resulted in structural instability and deterioration of the functional properties of the film. The mechanical analysis results further support this interpretation, indicating that the incorporation of 0.5% RVPH led to a certain improvement in mechanical properties, whereas increasing the protein hydrolysate content resulted in a gradual decline in mechanical strength.

However, despite exhibiting comparatively better mechanical performance than the other RVPH-containing formulations, the film containing 0.5% RVPH was ranked third overall. This outcome can be attributed to the influence of optical and water-related properties within the multi-criteria evaluation framework. These findings clearly demonstrate that determining the optimal formulation in multicomponent matrices cannot rely solely on a single performance parameter and highlight the necessity of multidimensional evaluation tools such as MCDM approaches.

Importantly, this outcome does not indicate that RVPH is ineffective as a functional additive; rather, it highlights the complex structure–property relationships governing multicomponent polysaccharide–protein film systems. The results demonstrate that the influence of protein hydrolysates on film performance is strongly dependent on concentration-controlled interactions within the polymer network, and that excessive incorporation may disturb the delicate balance of intermolecular forces responsible for stabilizing the matrix structure.

Nevertheless, among the RVPH-containing formulations, A3 (1% RVPH) was identified as the most appropriate alternative, as its mechanical and overall performance characteristics were closer to those of the control matrix compared with both lower and higher incorporation levels.

## 4. Conclusions

In this study, a multi-component composite film system based on alginate, chitosan, κ-carrageenan, agar, and *Rapana venosa* protein hydrolysate (RVPH) was successfully developed, and the structural role of RVPH within this polysaccharide matrix was systematically elucidated. The results demonstrated that RVPH did not induce chemical modification of the polysaccharide backbone; rather, it interacted with the matrix mainly through non-covalent interactions. FTIR and SEM analyses indicated that RVPH participated in hydrogen bonding and electrostatic interactions within the existing polyelectrolyte complex, contributing to concentration-dependent reorganization of the polymer network and influencing microstructural continuity and surface morphology.

All formulations exhibited high swelling capacity and water solubility due to the hydrophilic nature of the constituent polymers. The incorporation of RVPH modulated optical and mechanical properties in a non-linear manner. In particular, low incorporation levels caused partial disruption of the polymer network, whereas intermediate concentrations enabled partial structural reorganization within the matrix. Multi-criteria decision-making (MCDM) analysis identified 1% RVPH as the optimal incorporation level, providing improved opacity and reduced light transmittance while maintaining acceptable structural integrity.

From a materials design perspective, these findings clarify the role of marine protein hydrolysates in multicomponent polysaccharide films. In bio-based food packaging systems, film performance generally arises from two complementary strategies: enhancing the structural integrity of the polymer matrix, which may also contribute to improved barrier performance against moisture and gases, or incorporating functional additives that provide bioactive properties relevant to food preservation. The present results indicate that RVPH primarily acts as a structural modifier interacting with the polymer network rather than as a reinforcing component in this specific matrix system.

Overall, the study provides insight into the compatibility and structure–property relationships of RVPH in polysaccharide-based composite films and demonstrates how concentration-dependent interactions influence film organization. These findings contribute to the rational design of biodegradable film systems in which the balance between structural stability, optical shielding, and potential functional performance must be carefully controlled. Although the present work focuses on the structural, optical, and mechanical characteristics of the film matrix, the results provide a foundation for future studies investigating barrier properties (e.g., water vapor and oxygen permeability) and their relationship with food preservation performance in practical food lining applications under real storage conditions.

## Figures and Tables

**Table 1:** Composition of film formulations used in the study.

Component	Control Film (F0)	F1 (0.5% RVPH)	F2 (1.0% RVPH)	F3 (1.5% RVPH)
Chitosan (% *w*/*w*)	2.0	2.0	2.0	2.0
Agar (g)	0.3	0.3	0.3	0.3
κ-Carrageenan (g)	0.15	0.15	0.15	0.15
Sodium alginate (% *w*/*v*)	0.5	0.5	0.5	0.5
RVPH (% *w*/*w*)	0	0.5	1	1.5
Glycerol (% *w*/*w* of total polymer)	2.5	2.5	2.5	2.5
Casting volume (mL)	23	23	23	23

**Table 2:** The List of Criteria and Alternatives.

**Codes**	**Criteria**
C1	Mechanical performance
C2	Physical stability
C3	Water resistance/Structural integrity
C4	Transparency
**Codes**	**Alternatives**
A1	0 RVPH
A2	0.5 RVPH
A3	1 RVPH
A4	1.5 RVPH

**Table 3:** Mean film thickness values (mm) of RVPH-incorporated formulations.

Film Formulations	Thickness (Average)
0 RVPH	0.1175 ^a^
0.5 RVPH	0.0955 ^b^
1 RVPH	0.0740 ^c^
1.5 RVPH	0.1085 ^d^

Different superscript letters within the same column indicate significant differences (*p* < 0.05) according to one-way ANOVA followed by Tukey’s multiple range test.

**Table 4:** Effect of RVPH concentration on swelling ratio and water solubility of composite films.

Film Formulations	Swelling Ratio (g/g)	Water Solubility (%)
0 RVPH	7 ^a^	87.5 ^a^
0.5 RVPH	0.25 ^b^	73.5 ^b^
1 RVPH	0.375 ^b^	90 ^c^
1.5 RVPH	0.5 ^b^	92.3 ^d^

Different superscript letters within the same column indicate significant differences (*p* < 0.05) according to one-way ANOVA followed by Tukey’s multiple range test.

**Table 5:** Color properties of films.

Film Formulations	L*	a*	b*
0 RVPH	35.42 ^a^	0.01 ^a^	−0.40 ^a^
0.5 RVPH	35.71 ^a^	−0.01 ^b^	−0.46 ^b^
1 RVPH	36.00 ^a^	−0.12 ^c^	0.24 ^c^
1.5 RVPH	36.2 ^a^	−0.13 ^d^	0.90 ^d^

Different superscript letters within the same column indicate significant differences (*p* < 0.05) according to one-way ANOVA followed by Tukey’s multiple range test.

**Table 6:** Optical properties of films.

Film Formulations	Opacity (mm^−1^)	Transmittance (%)
0 RVPH	2.44 ^a^	51.6 ^a^
0.5 RVPH	3.63 ^b^	45 ^b^
1 RVPH	5.35 ^c^	40.01 ^c^
1.5 RVPH	3.00 ^d^	47.2 ^d^

Different superscript letters within the same column indicate significant differences (*p* < 0.05) according to one-way ANOVA followed by Tukey’s multiple range test.

**Table 7:** Mechanical properties of composite films containing different concentrations of protein hydrolysate.

Film Formulations	Tensile Strength (N/mm^2^)	Elongation at Break (%)	Toughness (MJ/m^3^)
0 RVPH	0.626 ± 0.135 ^a^	71.74 ± 5.98 ^a^	0.062 ± 0.017 ^a^
0.5 RVPH	1.035 ± 0.440 ^a^	60.50 ± 2.00 ^a^	0.089 ± 0.053 ^a^
1 RVPH	0.601 ± 0.206 ^a^	67.01 ± 6.38 ^a^	0.056 ± 0.033 ^a^
1.5 RVPH	0.766 ± 0.342 ^a^	71.00 ± 5.56 ^a^	0.086 ± 0.055 ^a^

Values are expressed as mean ± standard deviation (*n* = 3). The same superscript letter (a) within the same column indicates no significant difference among samples (*p* > 0.05).

**Table 8:** Calculation of the overall ranking.

	KV1	KV2	KV3	KV4	KV5	Geometric Mean
Mechanical performance	1	1	1	1	1	1
Physical stability	2	3	2	3	3	2.551
Water resistance/Structural integrity	3	2	3	2	2	2.352
Transparency	4	4	4	4	4	4

**Table 9:** Calculation of parameters (DM1).

Criterion Name	Order of Importance	*sj*	*kj*	*qj*	*wj*
Mechanical performance	1		1	1	0.277
Physical stability	2	0.05	1.05	0.952	0.264
Water resistance/Structural integrity	3	0.10	1.10	0.865	0.240
Transparency	4	0.10	1.10	0.787	0.218

**Table 10:** Overall ranking of criteria.

Criterion Name	Arithmetic Mean	Geometric Mean
Mechanical performance	0.296	0.296
Physical stability	0.242	0.242
Water resistance/Structural integrity	0.257	0.257
Transparency	0.204	0.204

**Table 11:** EDAS Method Decision Matrix.

	C1	C2	C3	C4
A1	1	5	5	1
A2	3.365865	2	3.565205	2
A3	4.373448	3	3.465724	3
A4	4.573051	4	2.626528	4
	3.328091	3.5	3.664364	2.5

**Table 12:** Average Positive Distance Matrix Table.

	C1	C2	C3	C4
A1	0	0.428571	0.364493	0
A2	0.01135	0	0	0
A3	0.314101	0	0	0.2
A4	0.374076	0.142857	0	0.6

**Table 13:** Average Negative Distance Matrix Table.

	C1	C2	C3	C4
A1	0.700	0.000	0.000	0.600
A2	0.000	0.429	0.0271	0.200
A3	0.000	0.143	0.0542	0.000
A4	0.000	0.000	0.283	0.000

**Table 14:** Evaluation Scores and ASi Table.

	Asi	Ranking
A1	0.868	1
A2	0.236	4
A3	0.324	3
A4	0.610	2

## Data Availability

The original contributions presented in this study are included in the article. Further inquiries can be directed to the corresponding author.
